# Genome-wide meta-QTL analyses provide novel insight into disease resistance repertoires in common bean

**DOI:** 10.1186/s12864-022-08914-w

**Published:** 2022-10-03

**Authors:** Asma Rahmanzadeh, Bahman Khahani, S. Mohsen Taghavi, Moein Khojasteh, Ebrahim Osdaghi

**Affiliations:** 1 Department of Plant Protection, School of Agriculture, Shiraz University, Shiraz, 71441-65186 Iran; 2 Department of Plant Genetics and Production, College of Agriculture, Shiraz University, Shiraz, Iran; 3 Department of Plant Protection, College of Agriculture, University of Tehran, Karaj, 31587-77871 Iran

**Keywords:** Antrachnose, Halo blight, Meta-analysis, *Phaseolus vulgaris*, Quantitative trait loci, White mold

## Abstract

**Background:**

Common bean (*Phaseolus vulgaris*) is considered a staple food in a number of developing countries. Several diseases attack the crop leading to substantial economic losses around the globe. However, the crop has rarely been investigated for multiple disease resistance traits using Meta-analysis approach.

**Results and conclusions:**

In this study, in order to identify the most reliable and stable quantitative trait loci (QTL) conveying disease resistance in common bean, we carried out a meta-QTL (MQTL) analysis using 152 QTLs belonging to 44 populations reported in 33 publications within the past 20 years. These QTLs were decreased into nine MQTLs and the average of confidence interval (CI) was reduced by 2.64 folds with an average of 5.12 cM in MQTLs. Uneven distribution of MQTLs across common bean genome was noted where sub-telomeric regions carry most of the corresponding genes and MQTLs. One MQTL was identified to be specifically associated with resistance to halo blight disease caused by the bacterial pathogen *Pseudomonas savastanoi* pv. *phaseolicola*, while three and one MQTLs were specifically associated with resistance to white mold and anthracnose caused by the fungal pathogens *Sclerotinia sclerotiorum* and *Colletotrichum lindemuthianum*, respectively. Furthermore, two MQTLs were detected governing resistance to halo blight and anthracnose, while two MQTLs were detected for resistance against anthracnose and white mold, suggesting putative genes governing resistance against these diseases at a shared locus. Comparative genomics and synteny analyses provide a valuable strategy to identify a number of well‑known functionally described genes as well as numerous putative novels candidate genes in common bean, *Arabidopsis* and soybean genomes.

**Supplementary Information:**

The online version contains supplementary material available at 10.1186/s12864-022-08914-w.

## Introduction

Common bean (*Phaseolus vulgaris*) is a major source of dietary protein in many developing countries and considered the most cultivated food legume for direct consumption around the world [1]. Thanks to high diversity in common bean varieties *i.e.* pinto, kidney, haricot, navy and Mexican, the crop is produced in different environmental conditions ranging from sub-Saharan Africa to northern Europe and from Latin America to Canada, as well as Eastern Asia [2]. Since the beginning of the current century, there has been a fast-growing trend in the production and consumption of common bean *i.e.* dry bean and/or green bean around the world. According to FAOSTAT [3], global production of dry beans and green beans in 1997 was 16.29 and 8.90 million tonnes (mt), respectively. By 2021, the value was gradually increased over time and reached to 27.54 and 23.27 million tonnes (mt) for dry bean and green bean production, respectively. Such a substantial growth in the commercial production of common bean had a concomitant increase of crop loss risks due to abiotic and biotic constraints [4]. Besides the environmental factors, *e.g.* soil quality, precipitation, and temperature, diseases caused by bacterial, fungal and viral agents have frequently been reported to affect common bean productivity around the globe [5, 6].

Bacterial diseases of common bean include common bacterial blight caused by *Xanthomonas phaseoli* pv. *phaseoli* (formerly known as *X. axonopodis* pv. *phaseoli*), halo blight caused by *Pseudomonas savastanoi* pv. *phaseolicola*, and bacterial wilt caused by *Curtobacterium flaccumfaciens* pv. *flaccumfaciens* [5, 7, 8]. White mold caused by *Sclerotinia sclerotiorum*, anthracnose caused by *Colletotrichum lindemuthianum*, and root rot disease complex caused by a consortium of fungal species *i.e. Fusarium solani* f.sp. *phaseoli*, *Fusarium oxysporum* f.sp. *phaseoli*, *Macrophomina phaseolina*, and *Rhizoctonia solani* are considered the most important fungal diseases of common bean. As for the viral agents, Bean common mosaic virus (BCMV), Bean golden yellow mosaic virus (BGYMV), and Bean golden mosaic virus (BGMV) are considered the most important pathogens of common bean [5]. Severe epidemics of these individual diseases have frequently been reported in different corners of the globe leading to significant yield losses. However, the main economic impact of biotic constraints in common bean industry is usually due to the occurrence of a combination of bacterial, fungal and viral diseases coupled with physiological disorders and unfavourite environmental conditions [4].

The use of certified disease-free seed lots and timely application of agrochemicals *e.g.* fungicides are recommended to combat the risk of bacterial and fungal diseases of beans [9, 10]. However, a sustainable and durable approach should simultaneously target all biotic constraints *i.e.* bacterial, fungal and viral pathogens to reduce the effort and labor of disease management procedure. Development of resistant cultivars against the above-mentioned pathogens not only reduces the cost needed for disease management also increases the profitability of cropping systems within the framework of sustainable agriculture [11]. However, pathogens frequently adapt themselves to the genetic background of the host plants and could overcome host resistance particularly when it is determined by major genes or R genes (resistance genes), which also known as qualitative resistance [12]. Quantitative resistance, on the other hand, which is governed by multiple genes, has a partial effect and is difficult to be disrupted by the pathogen; thus, gained interest in recent decades to address the major challenge of genetic resistance durability. A quantitative trait locus (QTL) is a region of DNA, which is associated with a particular phenotypic trait, and identifies chromosomal regions associated with significant genetic effects for traits of interest [13, 14]. Because QTLs are important for providing broad-spectrum and long-lasting resistance to pathogens, identifying and transferring QTLs into cultivars with appropriate agronomic features is a powerful way to develop a superior cultivar with a combination of agronomically desired traits and disease resistance [15]. Identification of QTLs conferring resistance against plant diseases, validation of their chromosomal position, and characterization of the corresponding genomic markers are important for breeders to execute marker assisted selection (MAS) programs [16, 17]. However, individual QTLs reported in the literature are associated with particular genotypes and environmental conditions and in the cases of diseases; they depend on the genotype of the pathogen in different experiments [16, 17, 18, 19]. It is necessary to identify and investigate cultivars containing resistance genes conferring resistance against multiple races of pathogens [20].

When it comes to breeding crops for multiple disease resistance traits, Meta-QTL (MQTL) analysis would be the approach of choice to collect all QTLs that contribute to resistance mechanisms. MQTL analysis is a statistical method that combines results from different independent studies for removing redundant QTLs and detecting consistent QTLs [21, 22]. This method was initially proposed by Goffinet and Gerber [23], and then improved by Veyrieras et al. [24]. MQTL analysis could recommend the stable QTLs from different studies, and at the same time reduces the confidence interval (CI) of QTL [25, 26, 27]. Meta-analysis of QTLs is applied to compilation of data from independent mapping populations [27, 28]. This approach has been used to find MQTL for crop disease resistance including *Fusarium* head blight in wheat [29], *Phytophthora*-related diseases in cocoa [30], cyst nematode in soybean [31], white mold [13] and anthracnose [20] in common bean. Furthermore, there are several studies describing multiple disease resistance meta-QTLs (MDR-MQTLs) conveying resistance against different types of pathogens in different crops [32, 33, 34, 35, 36]. For instance, Pal et al. [32] recently investigated the meta-QTLs and candidate genes for multiple disease resistance against leaf rust, stem rust, and yellow rust in wheat.

No MDR-MQTLs analysis has so far been conducted to identify major QTLs contributing to disease resistance in common bean. Hence, the main objective of the present study was to evaluate 152 QTL data in common bean reported in the literature to provide a comprehensive overview on disease resistance repertories in this crop. The results obtained in this study provide a better understanding of disease resistance mechanisms in common bean and at the same time pave the way of developing novel high profitable common bean cultivars with higher level of resistance against diverse diseases.

## Materials and methods

### QTLs used for MQTL analysis

The QTLs for disease resistance-associated features in common bean genome published within the past 20 years (up to 2021) were collected from the literature to be used in MQTL analysis. In order to identify the most stable genomic regions governing disease resistance in common bean, the QTLs lacking proper genetic map or QTL-related information were ignored. Table 1 presents QTL data used in this study including marker type, as well as population type, size, and parents. The mapping populations ranged in size from 52 to 907 progenies of various types including two backcross (BC), seven F2, and 35 recombinant inbred lines (RIL). Eventually, based on the availability and abundance of the QTL data, two bacterial diseases *i.e.* common bacterial blight and halo blight, and four fungal diseases including white mold, *Fusarium* root rot, anthracnose, and angular leaf spot were included in MQTL analyses.

**Table 1:** Summary of QTL studies used in Meta-QTL analysis of common bean resistance against common bacterial blight (CBB), halo blight (HB), white mold (WM), *fusarium* root rot (FRR), anthracnose (ANT) and angular leaf spot (ALS) diseases

**Population**	**Parents of population**	**Population size**	**Population type**	**Marker Types**	**Traits**	**References**
Pop1	Dorado× XAN 176	79	RIL	RAPD, SCAR, and ISSR	CBB	[37]
Pop2	OAC × OAC 95–4	142	F2	RAPD, AFLP, SSR, and SCAR	CBB	[38]
Pop3	HR67 × OAC95-4	112	RIL	SSR	CBB	[39]
Pop4	CHW × CHC	221	F2	AFLP	CBB	[40]
Pop5	HR67 × OAC95-4	81	RIL	SSR, SCAR, STS, and AFLP	CBB	[41]
Pop6	HR45 × OAC Rex	907	F2	SSR, SCAR, STS, and AFLP	CBB	[41]
Pop7	XAN159 × PI319443	81	RIL	STS	CBB	[42]
Pop8	OAC Rex × HR45	218	RIL	SSR, SNP, and SCAR	CBB	[43]
Pop9	Long 5 × Long 4	803	F2	SSR	CBB	[44]
Pop0	Sanilac × OAC 09–3	90	RIL	SSR, SNP, and SCAR	CBB	[45]
Pop11	Jules × Canela	103	F2	RAPD and AFLP	HB	[46]
Pop12	Wonder × UI-3	52	RIL	RAPD and SCAR	HB	[47]
Pop13	ZAA 12 × Canadian Wonder	79	RIL	RAPD and SCAR	HB	[48]
Pop14	BelNeb-RR-1 × A55	76	RIL	SNP	HB	[49]
Pop15	Minuette × OSU5630	77	RIL	SNP	HB	[49]
Pop16	Xana × Cornell 49,242	110	RIL	SSR	HB	[14]
Pop17	PMB0225 × PHA1037	185	RIL	SNP and SSR	HB	[50]
Pop18	UI3 × Tendergreen	97	F2	AFLP and SSR	HB	[51]
Pop19	UI3 × A52	64	F2	AFLP and 3SSR	HB	[51]
Pop20	Bunsi × Newport	98	RIL	RAPD and AFLP	WM	[52]
Pop21	Huron × a C-20	28	RIL	RAPD and AFLP	WM	[52]
Pop22	Bunsi × Raven	98	RIL	AFLP and RAPD	WM	[53]
Pop23	Aztec × ND88–106–04	85	RIL	AFLP, SCAR, TRAP, and RAPD	WM	[54]
Pop24	G122 × CO72548	94	RIL	AFLP, microsatellites, RAPD, and SCAR	WM	[55]
Pop25	Tacana × PI 318,695	89	BC	SSR and SRAP	WM	[56]
Pop26	Tacana × PI 313,850	75	BC	SSR and SRAP	WM	[56]
Pop27	Xana × Cornell 49,242	104	RIL	AFLP	WM	[57]
Pop28	AN-37 × P02630	94	RIL	SNP	WM	[58]
Pop29	Orion × Orion/R31-83	104	RIL	SNP	WM	[13]
Pop30	Montrose × I9365-25	130	RIL	SNP	WM	[13]
Pop31	UI-537 × I9365-25	127	RIL	SNP	WM	[13]
Pop32	Orion × USPT-WM-12	158	RIL	SNP	WM	[13]
Pop33	A195 × OSU6137	114	RIL	SNP	WM	[13]
Pop34	G122 × WMG9-04–20-3	80	RIL	SNP	WM	[13]
Pop35	OSU5446 × RR6950	170	RIL	SNP	FRR	[59]
Pop36	Puebla 152 × Zorro	121	RIL	SNP	FRR	[60]
Pop37	CAL96 × MLB-49-89A	121	RIL	SNP	FRR	[61]
Pop38	JaloEEP558 × BAT93	77	RIL	RFLP and RAPD	ANT	[62]
Pop39	G19833 × DOR364	87	RIL	RFLP	ANT	[63]
Pop40	IAC-UNA × CAL 143	380	RIL	SSR	ANT	[64]
Pop41	DOR364 × G19833	87	RIL	SSR	ANT	[65]
Pop42	PMB0225 × PHA1037	185	RIL	SCAR, AFLP, SSR, and SNP	ANT	[66]
Pop43	G19833 × DOR364	87	RIL	RFLP	ALS	[67]
Pop44	IAC-UNA × CAL	346	RIL	SCAR and SSR	ALS	[68]

### QTLs projection and meta-analysis

Based on the inclusion of different types of markers and high saturation of markers, two reference maps of common bean genome were retrieved from the the “Legume Information System (LIS)” online service https://www.legumeinfo.org/, and subjected to QTL projection [31, 32]. The reference map of Galeano et al. [69] has different marker types including AFLP, RAPD, SNP, SSR and STS. It has 1,269 markers with an average distance of 1.60 cM between markers, with the average chromosome length being 185.58 cM for a total length of 2,041.45 cM. In the MQTL analysis, the studies that were based on different types of SNP markers were projected onto the Song et al. [70] map. The latter reference map has 3,564 SNP markers with an average distance of 0.29 cM, while the average chromosome length was 94.15 cM with a total length of 1,035.68 cM [70]. Hence, among the QTL studies mentioned in Table 1, those published by Hoyos-Villegas et al. [58], Nakedde et al. [60], Wang [61], and Vasconcellos et al. [13] were projected to Song et al. [70] map, while the remaining studies were projected to the Galeano et al. [69] map.

For each of the QTLs, genetic position, proportion of phenotypic variance (R2), confidence Interval (CI) and the log of odds ratio (LOD score) were considered in the subsequent analyses. In order to incorporate the QTLs lacking the CI data, we applied the formulas CI = 530/(N*R2) for BC and F2 lines, CI = 287/(N*R2) for DH lines and CI = 163/(N*R2) for RILs lines, where N is the population size and R2 is the proportion of phenotypic variance of the QTL [71]. Correlation analysis between the number of initial QTLs and projected QTLs was conducted for all chromosomes of common bean. Further, to conduct a meta-analysis BioMercator V4.2 [72, 73] was used to integrate QTLs on the two above-mentioned reference maps. Consequently, the prevalent value among Akaike information criterion (AIC), corrected AIC (AICc), AIC 3 candidate models (AIC3), Bayesian information criterion (BIC) and average weight of evidence (AWE) models for each chromosome was chosen and considered as the number of MQTLs. Subsequently, the SOFIA package implemented in R-studio was used to show the distribution of QTLs within each MQTL, as well as the MQTL density on the reference maps [74].

### Gene density and distribution of MQTLs

The data for gene density and variation distribution on common bean genome was retrieved from the EnsemblPlants (https://plants.ensembl.org/index.html) and Phytozome (https://phytozome-next.jgi.doe.gov/) databases, respectively, using RIdeogram via R-studio package [75]. Genomic position of predicted MQTLs and their distribution on each chromosome was determined using pheatmap R package and noticed on the 11 common bean chromosomes as a heatmap [76]. The position of MQTLs on common bean genome was compared to that of the gene density along each chromosome and illustrated using the pheatmap and RIdeogram R packages. Additionally, variations on the genome and the position of MQTLs among genotypes were presented using ChromoMap [77]. Sequences of flanking markers of MQTLs were obtained from the legumeinfo database (https://legumeinfo.org/search/marker), while the nearest markers to each MQTL were used to find the genomic position of MQTLs.

### Comparison of MQTLs with GWAS studies

In order to improve the accuracy of our investigations, the most significant loci derived from genome-wide association studies (GWAS) were retrieved and compared to the genomic position of MQTLs in common bean for white mold- and anthracnose-related characteristics. Hence, six GWAS studies were surveyed to find the co-located significant GWAS signals and near-significant GWAS signals with MQTLs based on the common bean genome [78, 79, 80, 81, 82, 83].

### Identification of Candidate Genes (CGs) in MQTL regions and KEGG enrichment analysis

The common bean genome in EnsemblPlants was used to identify disease resistance-related functional candidate genes (CGs) located at the CI of each detected MQTL. The sequences of all markers were used for BLAST analysis against common bean genome. EnsemblPlants database was used to extract gene annotations of the MQTL genomic regions (https://plants.ensembl.org/index.html). In order to improve the accuracy of our assumption on functional CGs, the orthologues of genes located at each MQTL interval were placed over the *Arabidopsis* (*Arabidopsis thaliana*) and soybean (*Glycine max*) genomes. This would lead to identifying more potential functional CGs based on their reported function in *Arabidopsis* and soybean. The Circos was used to present the gene density and orthologue genes in common bean, *Arabidopsis* and soybean [84]. Moreover, the KOBAS v3.0 software (http://kobas.cbi.pku.edu.cn/) was used to improve our understanding of the function of genes through KEGG pathways [85]. In order to conduct this analysis, the genes that were involved in MQTL regions were blasted against *Arabidopsis* genome through KOBAS v3.0 software. The ggplot2 package [86] was exploited to show the KEGG results.

### Association of MQTLs with known R genes

The relationship of MQTLs with known R genes for the common bean diseases was investigated [87]. To this aim, EnsemblPlants database was used to retrieve FASTA sequences of MQTL regions, which were then BLASTed against the plant disease resistance gene database (PRGDB) to find PR-proteins and resistance gene analogs. In order to improve the accuracy, those MQTL regions less than five Mb were considered for this analysis. Genomic position of predicted PR-proteins on each chromosome was represented using Mapchart V.2.32 software [88]. Subsequently, the expression of these genes was retrieved from (https://phytozome-next.jgi.doe.gov/) for different tissues, standardized through z-scores in each tissue, and finally illustrated by pheatmap package in R.

### Collection of datasets and analysis of gene expression

In order to validate the candidate genes (CGs) located on MQTL regions, four differentially expressed genes (DEGs) datasets involved in resistance to halo blight [51, 89], anthracnose [90], and white mold [91] were collected from independent studies. Venn diagram was used to compare the common bean resistance-responsive genes identified by four DEGs studies, and the genes located in MQTL regions. Candidate genes shared between MQTL regions, and DEGs data were analyzed in Venny online tool (https://bioinfogp.cnb.csic.es/tools/venny/index.html).

## Results

### Main features of resistance-related QTL studies in common bean

**Fig. 1:**
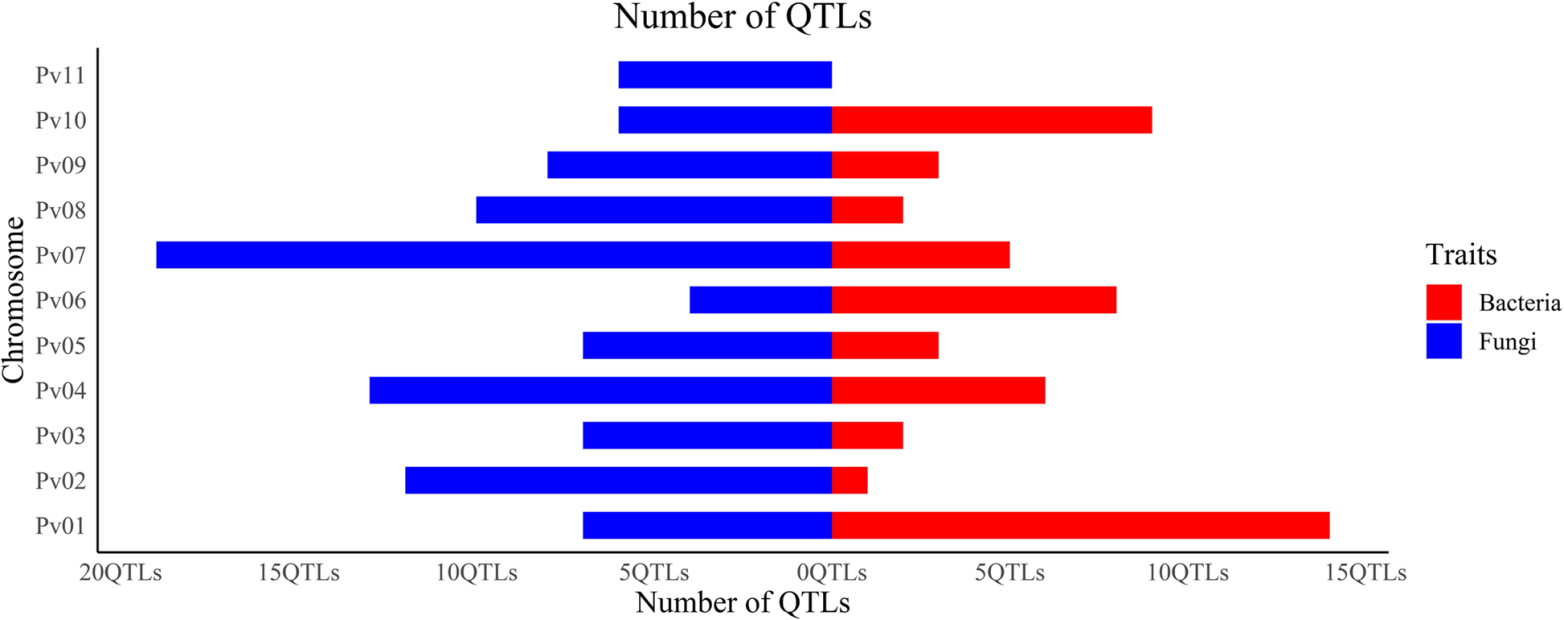
The highest number of QTLs governing resistance to fungal diseases in common bean was found on chromosome 7, while the highest number of QTLs governing resistance against bacterial diseases was located on chromosome 1

Data of 152 QTLs published between 2000 and 2021 were retrieved from the literature. These QTL studies included two BCs, seven F2s, and 35 RILs (Table 1). The number of QTLs responsible for resistance against bacterial and fungal diseases and their distribution on 11 common bean chromosomes are presented in Fig. 1. These QTLs belonged to 44 populations reported in 33 publications and contributed to common bean resistance against bacterial diseases *i.e.* common bacterial blight and halo blight, and fungal diseases *i.e.* white mold, *Fusarium* root rot, anthracnose and angular leaf spot. The QTLs were dispersed unevenly across the chromosomes where chromosome 11 with six QTLs and chromosome 7 with 24 QTLs had the lowest and highest number of QTLs, respectively (Fig. 1). Among the diseases investigated in this study, chromosome 7 had the highest number of resistance QTLs against fungal diseases while the highest number of QTLs conveying resistance against bacterial diseases was found on chromosome 1.

### QTLs projection and detected MQTLs

In order to improve the accuracy and the number of projected QTLs, the two common bean reference maps provided by Galeano et al. [69] and Song et al. [70] were used in this study. The former map contained 1,269 markers while the latter contained 3,560 markers. To avoid any inconsistency, the case studies or reported QTLs with inverted or undetermined positions – as compared to the reference map – were excluded from the subsequent analyses. For consensus QTL prediction, the chromosomal position, LOD score, and R2 of the individual QTLs were taken into consideration. Only 57 out of 152 QTLs belonging to one bacterial disease (halo blight) and four fungal diseases (anthracnose, white mold, angular lead spot, and *Fusarium* root rot) were projected on the reference maps. Among the 57 projected QTLs, 32 QTLs were identified as major (PVE˃ 10%, [92, 93]), while 25 QTLs were identified as minor (Table S1). Among 57 QTLs, nine MQTLs were identified in common bean genome where three MQTLs (33%) were retrieved from at least three independent studies (Table 2). In this study, we only report MQTLs that contained more than two QTLs. As a result, MQTL analysis narrowed down the confidence Interval (CI) of genomic regions governing disease resistance compared to that of QTL studies. Moreover, a low correlation between the number of initial QTLs and projected QTLs was inferred on common bean chromosomes (*r* = 0.40). A MQTL with higher number of initial QTLs is the more stable one regardless of genetic background or environmental factors. MQTL7P.1 located on chromosome 7 with five QTLs had the highest number of initial QTLs generated from four separate populations followed by MQTL2P.1 and MQTL9P.2 on chromosomes 2 and 9, respectively, with three initial QTLs generated from three different populations (Table 2). These MQTLs were the most robust, viable and stable QTLs in a wide geographical and temporal span.

**Table 2:** Detected MQTLs for resistance against bacterial (halo blight = HB) and fungal (anthracnose = ANT and WM = white mold) diseases in common bean

MQTL	Chr. No	Flanking markers	MQTL position (cM)	MQTL confidence interval (cM)	Genomic position of MQTL (Mbp)	Number of initial QTLs	Number of Populations	Mean LOD/Mean PVE	Involving Traits	Number of genes laying at the MQTL interval
MQTL1P.1	1	ss715639329_ss715649324	25.24	1.39	3.34 _3.41	2	2	3.95 / 14%	WM	11
MQTL2P.1	2	BMb259_BMb252	81.1	2.86	14.33_21.59	3	3	2.94 / 11.66%	ANT, WM	193
MQTL5P.1	5	g136_g1395	4.15	3.03	3.05_6.65	2	1	3 / 11.5%	ANT, HB	191
MQTL7P.1	7	ss715648636_ss715646351	24.56	0.03	1.81_3.38	5	4	5.32 / 18.8%	WM	181
MQTL7P.2	7	Bng204_g415	91.93	7.06	37.40_44.34	2	1	5.67 / 10%	ANT	540
MQTL8P.1	8	BMb445_BMd25	28.29	0.95	1.02_1.40	2	2	3 / 12%	ANT, HB	58
MQTL9P.1	9	ss715645176_ss715645103	4.35	0.03	7.70_8.74	2	1	3.75 / 12%	WM	90
MQTL9P.2	9	g2209_BM114	13.04	14.46	36.53_36.95	3	3	4.22 / 10.66%	ANT, WM	44
MQTL10P.1	10	g1661_g2600	15.6	16.35	28.99_40.69	2	2	3 / 8%	HB	583

*Chr* Chromosome, *LOD* Logarithm of the odds, *PVE* Phenotypic variation explained

**Fig. 2:**
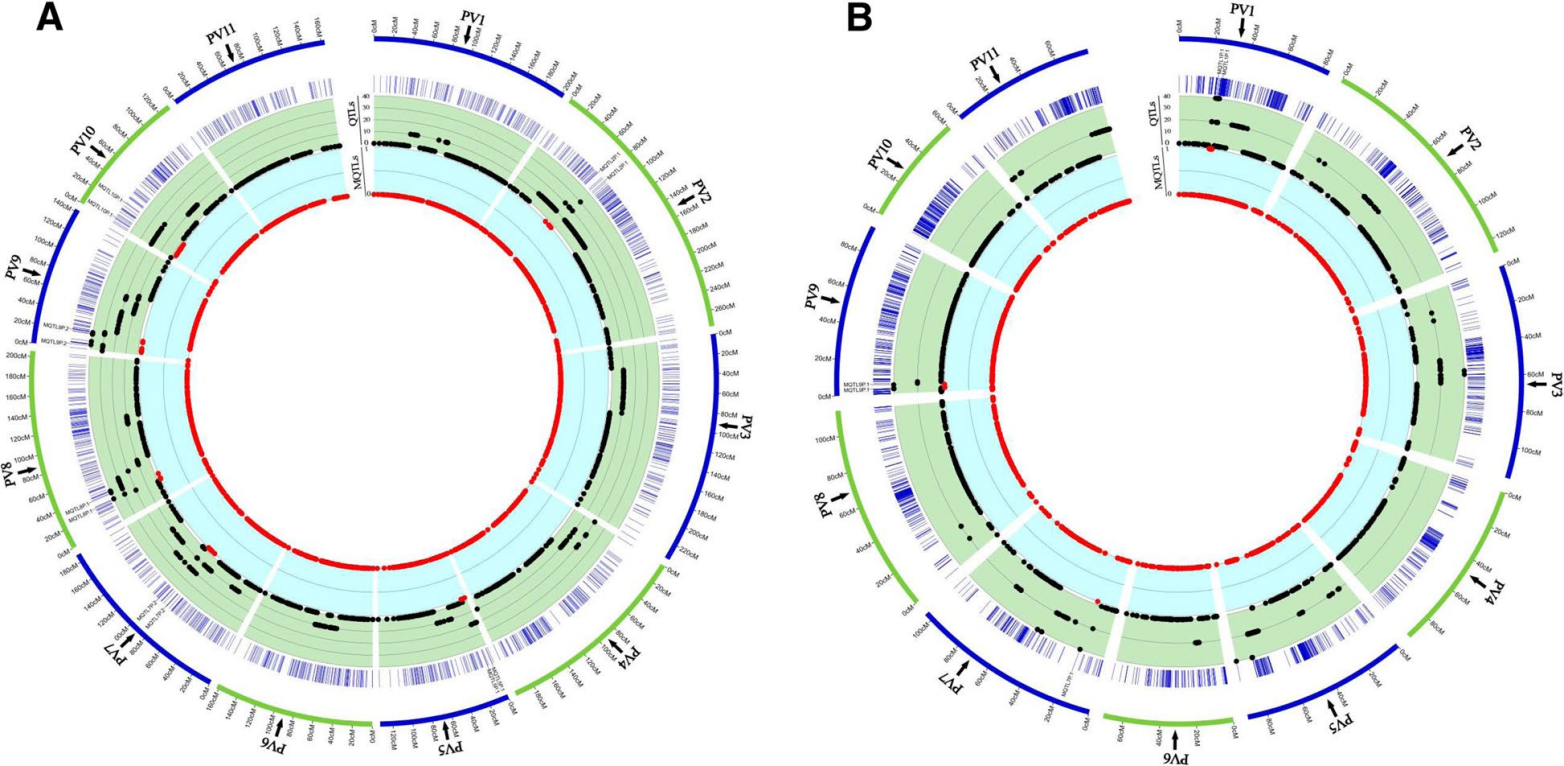
Distribution of QTLs and MQTLs on two common bean reference genome maps developed by Galeano et al. 2011 [69] (**A**) and Song et al. 2015 [70] **(B**) generated by SOFIA package in Rstudio [74]. The outermost circle shows the chromosomes’ position on the reference maps in cM. The second circle outlines the density of markers on the maps. The third circle displays the QTLs’ density (black dots) while the fourth circle displays the MQTLs density (red dots)

The MQTLs associated with resistance to each disease was irregularly scattered across common bean chromosomes where chromosomes 7 and 9 each had two MQTLs, chromosomes 1, 2, 5, 8 and 10 each had one MQTL, while no MQTL was found on the other chromosomes *i.e.* 3, 4, 6 and 11 (Fig. 2, Table 2). MQTL1P.1, MQTL7P.1 and MQTL9P.1 located on chromosomes 1, 7, and 9, respectively, represented the best chromosomal regions for resistance against white mold on chromosomes 1, 7 and 9, respectively. On the other hand, MQTL7P.2 on chromosome 7 had the highest number of QTLs associated with resistance against anthracnose. MQTL10P.1 on chromosome 10 showed region accommodating QTLs associated with halo blight resistance. Furthermore, four overlapping MQTLs including MQTL2P.1, MQTL5P.1, MQTL8P.1, and MQTL9P.2 located on chromosomes 2, 5, 8, and 9, respectively, that involved in resistance against at least two diseases were found. For instance, the two overlapping MQTLs *i.e.* MQTL5P.1 and MQTL8P.1 located on chromosomes 5 and 8, respectively, were observed to convey resistance against both bacterial (halo blight) and fungal (anthracnose) diseases of common bean on chromosomes 5 and 8, respectively. Hence, this region might contain genes controlling either types of infection or pleiotropic effects. Further details on the MQTLs flanking markers, their position, CI, and the traits which were controlled by MQTLs are reported in Table 2.

### MQTL distribution on common bean genome

**Fig. 3:**
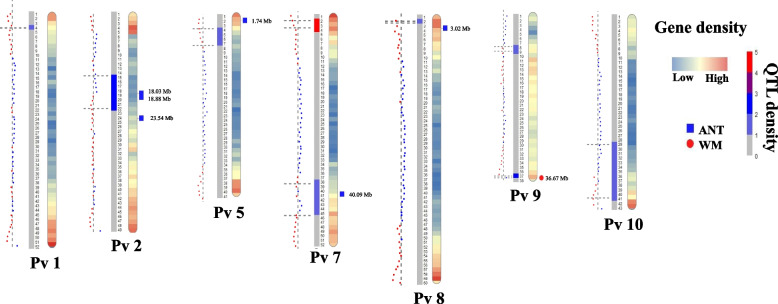
Distribution of MQTLs conveying resistance against bacterial and fungal diseases on common bean genome (95% confidence interval). Position of the MQTLs on genome is represented in Mb. In each pair of columns, gene density is shown in the inner of chromosome on the right side of the heatmap. The plot of variations is represented on the left part of the chromosome using a scatter plot in which the average of variations for each MB on each chromosome is indicated through the reference line. Additionally, the regions having variations more than average are presented with red dots. The position of MQTLs is highlighted with grid lines on each chromosome. The significant loci from genome-wide association studies (GWAS) with the genomic position in Mb are shown on the chromosomes for anthracnose and white mold

**Fig. 4:**
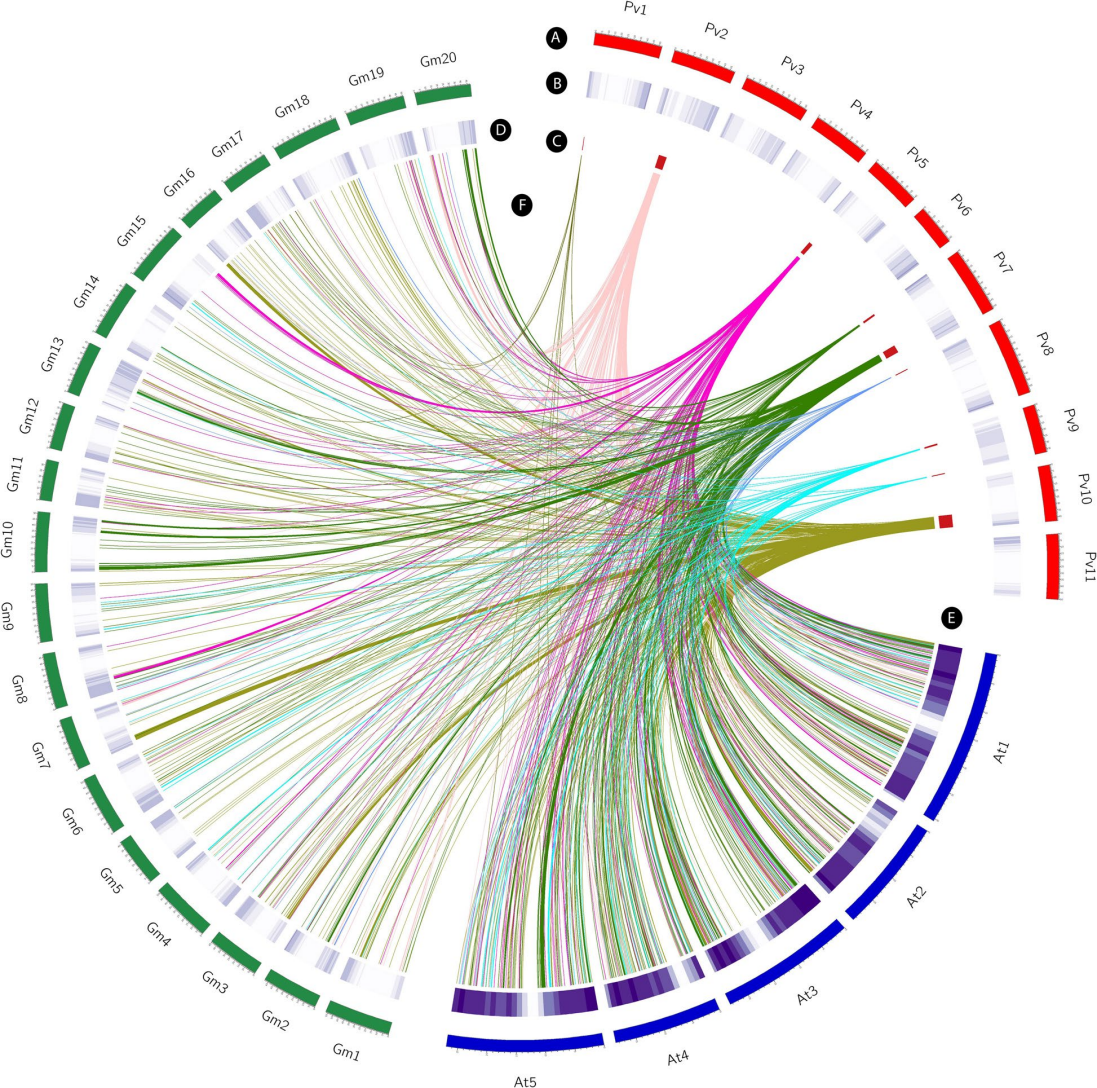
Circular map constructed using the Circos software showing the syntenic regions among common bean, soybean, and *Arabidopsis* genomes. A: common bean chromosomes, B: gene density on common bean chromosomes, C: position of MQTLs in common bean genome, D: gene density on soybean chromosomes, E: gene density on *Arabidopsis* chromosomes, F: common bean syntenic regions with soybean and *Arabidopsis*. The outermost circle represents the genomes in Mb

Distribution and genomic position of all MQTLs was determined on common bean genome based on the genomic location of flanking markers compared to gene density and variations existed in the genotypes. The gene density of all common bean chromosomes was retrieved from the EnsemblPlants database (Fig. 3). Gene density analyses showed that a majority of genes were located in sub-telomeric regions of the common bean chromosomes (Fig. 3). Similarly, most of the detected MQTLs were mainly located at the sub-telomeric regions where the variations were also higher for most MQTLs (Fig. 3). Comparison of resulted MQTLs with the significant loci associated with white mold and anthracnose in GWAS studies indicated that three MQTLs including MQTL2P.1, MQTL7P.2 and MQTL9P.2 respectively on chromosomes 2, 7 and 9 were in congruence with the GWAS results (Fig. 3). Additionally, three significant GWAS signals were detected near derived MQTLs (Fig. 3). This result shows the adaptability and coherence of gene density and MQTLs. Considering the synteny (physical co-localization of genetic loci on the same chromosome) among common bean, *Arabidopsis* and soybean genomes, the corresponding CGs of MQTLs identified in this study were investigated on *Arabidopsis* and soybean genomes. A schematic illustration of the CGs distribution in common bean, *Arabidopsis* and soybean genomes is shown in Fig. 4. This comparative genomics analysis provided a valuable approach for detecting the functional CGs and transferring information across species.

### CGs for disease resistance

MQTL analysis reduced the CI value up to 2.64 folds with the average of 5.12 cM in MQTLs compared to the mean of projected QTLs. In three of the MQTLs *i.e.* MQTL7P.1, MQTL8P.1 and MQTL9P.1 located on chromosomes 7, 8 and 9, respectively, the CI was reduced to < 1 cM (Table 2). Hence, the probability of exploration of the functional CGs in the interval of MQTLs is higher than that in QTLs. The list of 1,891 genes underlying the CI of each MQTL region on common bean genome along with their annotations and orthologue genes in *Arabidopsis* and soybean is presented in Tables S2, S3 and S4, respectively. A number of functionally important genes were identified to be associated with MQTL in common bean according to their role in *Arabidopsis* genome. For instance, orthologue of the *Arabidopsis* genes *RPS4* in common bean is *PHAVU_002G092000g* on MQTL2P.1 (chromosome 2), the orthologue of *Arabidopsis* gene *ZAR1* in common bean is *PHAVU_007G032100g* on MQTL7P.1 (chromosome 7), and the orthologue of *Arabidopsis* gene *VBF* in common bean is *PHAVU_007G198400g* on MQTL7P.2 (chromosome 7). Further, the orthologue of *Arabidopsis* genes *WRKY46* in common bean is *PHAVU_010G111900g* on MQTL10P.1 (chromosome 10). All the latter four genes are associated with defense mechanisms against plant diseases.

**Fig. 5:**
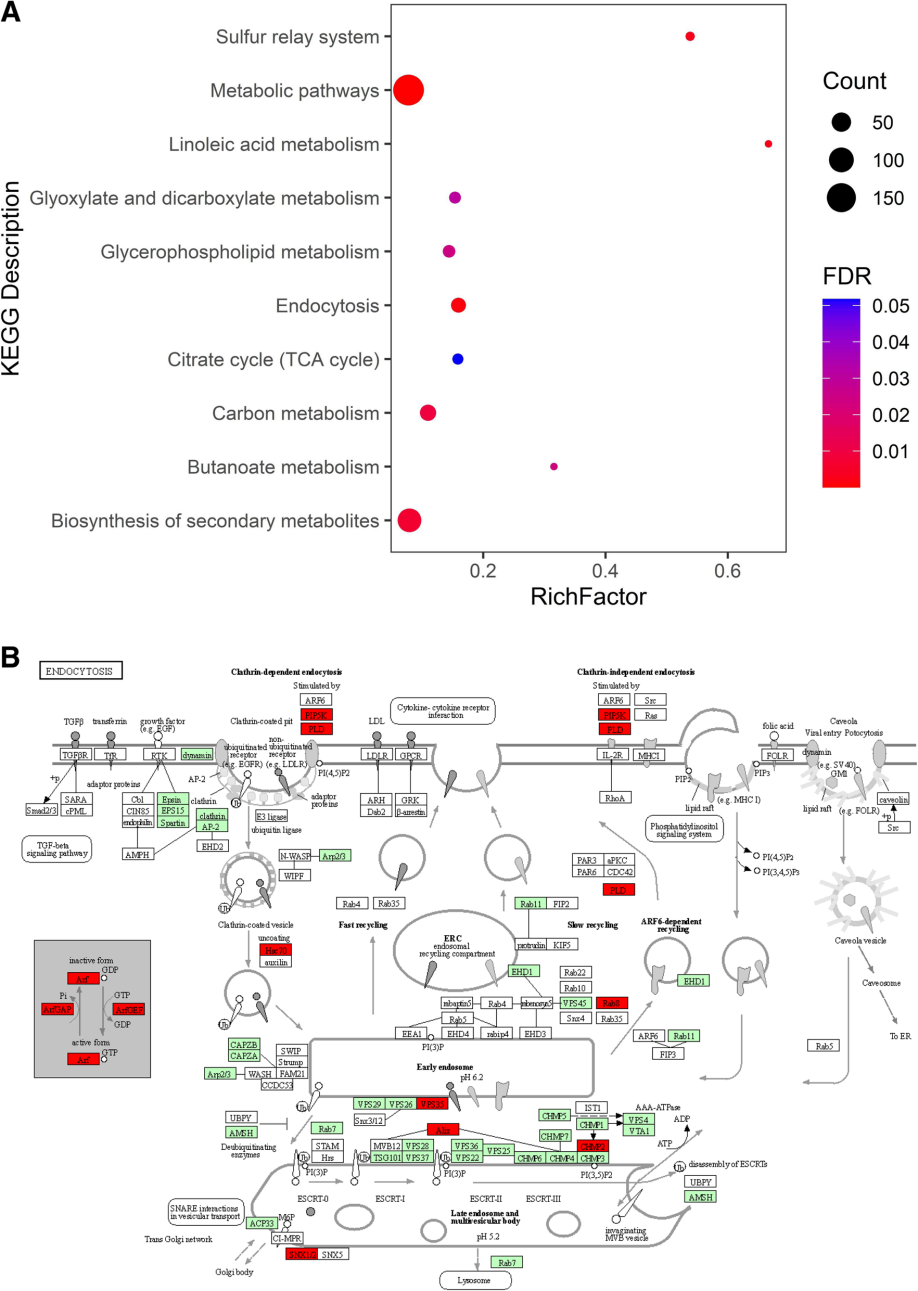
KEGG enrichment analysis of genes underlying MQTLs associated with resistance to bacterial and fungal disease in common bean (**A**). The y-axis represents the enriched KEGG pathways. The color and the size of pathways descriptions represent the FDR and the count, respectively. Rich factor on the x-axis is the ratio of the gene number to the total gene number in that specific pathway. **B** Endocytosis pathway map which is involved significantly in plant defense mechanisms against different pathogens. Red boxes represent genes identified in our MQTLs interval. The genes in the green background represent genes that have been previously identified in *Arabidopsis*. For additional information on the genes for each KEGG description of MQTLs see Table S6. Permission is granted to BMC Genomics to publish under the CC BY 4.0 open access license [59]

Subsequently, a biological pathway enrichment analysis was conducted on the CGs located at the CI of detected MQTLs. This would improve the understandings of the pathways controlling defense mechanisms of common bean against biotic stresses (Fig. 5a). One of the most noticeable mechanisms was endocytosis pathway (absorbing substances into the cell) which is involved significantly in plant defense mechanisms against different pathogens (Fig. 5b). Further details on the genes involved in the KEGG enrichment analysis are reported in Table S5.

### MQTLs carrying R genes

**Fig. 6:**
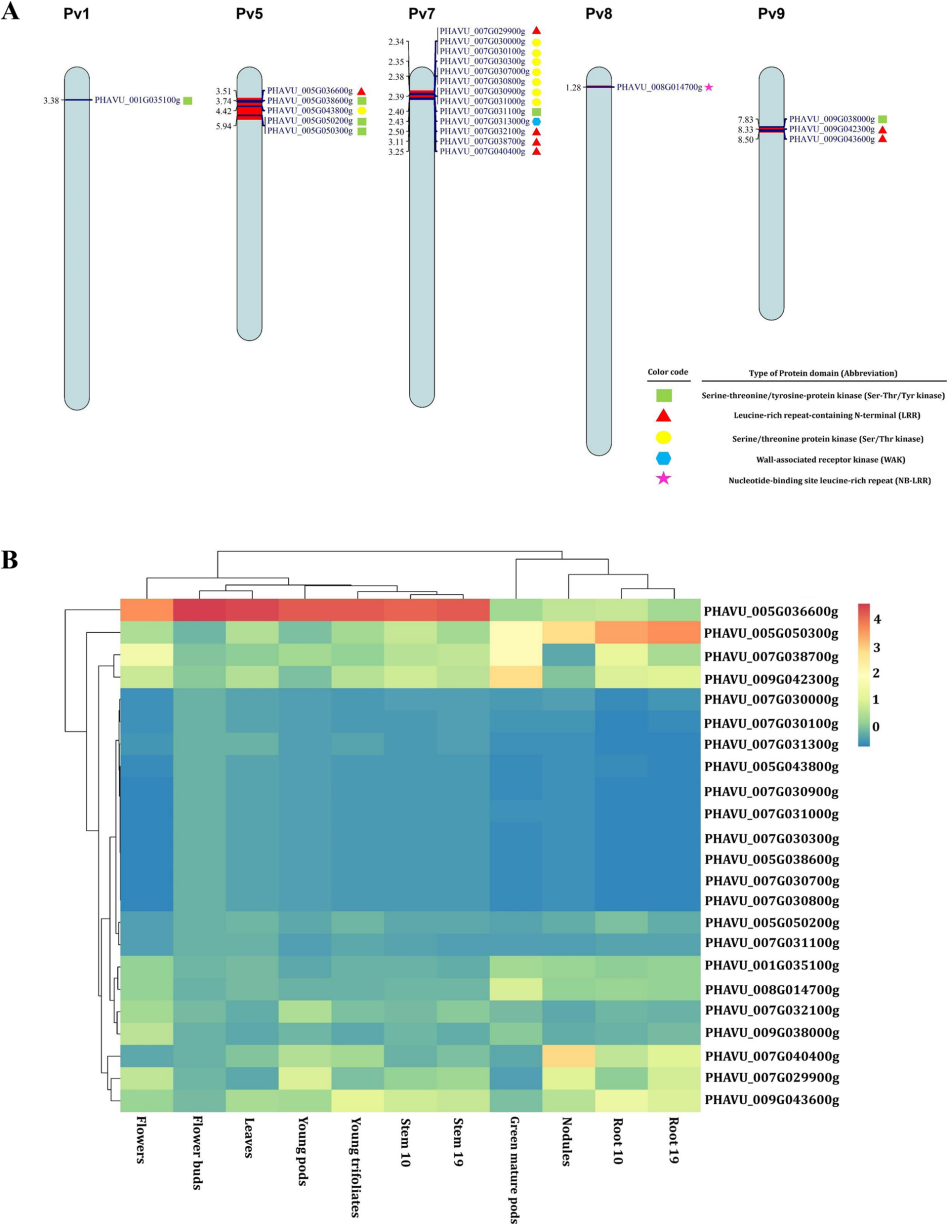
The predicted resistance genes (R gene) in MQTL regions associated with resistance to bacterial and fungal diseases in common bean. **A** The genomic position of predicted R genes within MQTL regions was represented on each chromosome. Different types of R genes domains were displayed by color codes in the bottom right-hand corner. **B** The expression of R genes identified within MQTL regions were displayed by a heat map across different tissues of the common bean

Based on the BLAST search against PRGdb, five MQTL regions were detected carrying resistance gene homologues (Fig. 6a and Table S6). A majority of the detected genes contained kinase domain, although some genes *i.e. PHAVU_005G036600g* (on MQTL5P.1 located on chromosome 5); *PHAVU_007G029900g*, *PHAVU_007G032100g*, *PHAVU_007g038700g*, and *PHAVU_007G040400g* on MQTL7P.1 (chromosome 7), as well as *PHAVU_009G042300g* and *PHAVU_009G043600g* on MQTL9P.1 (chromosome 9) contained leucine-rich repeat (LRR) domain. Notably, one gene (*PHAVU_008G014700g*) found on MQTL8P.1 (chromosome 8) included nucleotide-binding site leucine-rich repeat (NB-LRR) domain implicated in plant defense interactions (Fig. 6a). Furthermore, the gene expression pattern in different common bean tissues revealed that PR-proteins are expressed most strongly in flower buds, young trifoliates, and leaves, respectively. The *PHAVU_005G036600g* gene, located on MQTL5P.1, had the greatest expression in various tissues among the 23 genes found in our analyses (Fig. 6B).

### Common Candidate Genes (CGs) in MQTL regions and DEGs datasets

Resistance-responsive genes in common bean were collected from four independent studies. Based on DEGs data analysis, 1,805 DEGs were observed in response to different diseases including 293 DEGs for halo blight, 1,470 DEGs for anthracnose, and 42 DEGs for white mold. In total, 137 shared genes were detected between the DEGs studies and the genes located in MQTL regions using the Venn diagram (Figure S1, Table S7). The genes that are differentially expressed in response to diseases in common bean and located within MQTL regions can be considered as differentially expressed candidate genes (DECGs).

## Discussion

Reliable management of biotic constraints in common bean production depends on proper combination of integrated pest management (IPM) strategies *i.e.* host resistance, cultural practices, biological control, as well as chemical applications [15, 94]. In this study, we employed a genome-wide meta-analysis approach to evaluate QTL repertories of common bean for resistance against bacterial and fungal diseases. Uneven distribution of QTLs across common bean genome was observed with the lowest number of QTLs on chromosome 11 (*n* = 6) and the highest number on chromosome 7 (*n* = 24). Chromosome 7 had the highest number of resistance QTLs against fungal disease, while the highest number of QTLs governing resistance against bacterial diseases was found on chromosome 1. Out of 152 QTLs from 44 QTL populations, 57 QTLs were successfully projected on two common bean reference maps. Although MQTL analyses on common bean has already been conducted on the amount of iron and zinc content [95], resistance to anthracnose [20], and white mold [13], to the best of our knowledge, this is the first comprehensive multiple disease resistance meta-QTLs (MDR-MQTLs) analysis to decipher disease resistance-associated repertories in the crop.

Three MQTLs were detected conferring resistance to the bacterial disease halo blight (caused by *P. savastanoi* pv. *phaseolicola*), while six MQTLs were associated with resistance against each of the fungal diseases white mold and anthracnose caused by *Sclerotinia sclerotiorum* and *Colletotrichum lindemuthianum*, respectively. We detected a low correlation between QTLs and MQTLs across the chromosomes. MQTL analysis can detect stable QTLs regardless of their genetic background, chronology and location in the corresponding original investigation [16, 26, 30, 96]. In comparison to the MQTLs reported in the previous study on white mold [13] and anthracnose [20], we identified one further overlapping MQTL named MQTL7P.1 located on chromosome 7 conveying resistance against white mold disease. Four MQTLs (MQTL1P.1, MQTL5P.1, MQTL7P.1, MQTL8P.1) in our study overlapped with five MQTLs of anthracnose disease [20], three of which located at the closest region to previous study. Furthermore, four MQTLs including MQTL2P.1, MQTL5P.1, MQTL8P.1, MQTL9P.1 located at the same regions with the hotspot of QTLs in previous study on anthracnose [20]. Five novel MQTLs were also identified in this study conveying resistance against bacterial and fungal diseases. Investigation on the distribution of MQTLs is a progressive approach in crop breeding while our study is the first one in this regard on common bean genome. Our analyses indicated that sub-telomeric regions contain most of the detected MQTLs, gene density, and variations (Fig. 3). Interestingly, previous studies in rice, maize and barley showed similar pattern where higher densities of MQTLs and genes observed at sub-telomeric regions [26, 27, 28, 96]. One of the advantages of MQTL analysis is that it significantly narrows down the CI improving the precision of finding CGs in MQTL regions [26, 27, 28, 95, 97, 98, 99, 100]. In this study, MQTL analysis reduced the average of CI up to 2.64 folds with an average of 5.12 cM in MQTLs compared to the mean of projected QTLs where 1,891 genes were detected on interval regions of studied MQTLs.

Two shared MQTLs *i.e.* MQTL5P.1 and MQTL8P.1 located on chromosomes 5 and 8, respectively, were detected conveying resistance against halo blight and anthracnose while the two shared MQTLs MQTL2P.1 and MQTL9P.2 on chromosomes 2 and 9, respectively, were associated with resistance against anthracnose and white mold. The corresponding genes of these MQTLs were detected on *Arabidopsis* genome where they are involved in a number of functionally important pathways. Hence, they could be considered in plant breeding efforts to develop new lines with resistant to fungal and bacterial diseases. Orthologue of *PBL* gene family was detected on MQTL2P.1. Genes of the latter family in *Arabidopsis* are involved in resistance against bacterial black rot of crucifers caused by *Xanthomonas campestris* pv. *campestris* in the resistant ecotype Col-0. *PBL2* a serine/threonine-protein kinase involved in pathogen-associated molecular pattern (PAMP)-triggered immunity (PTI) signaling and defense responses downstream of flagellin sensing2 (*FLS2*) for plants. Additionally, *PBL2* is the strongest interactor of XopAC which is a major avirulence gene of *X. campestris* pv. *campestris* [101, 102]. Moreover, two orthologues of *WRKY3* and *WRKY4* are located on MQTL2P.1. The WRKY transcription factors play a vital role in pathogen-triggered signal transduction cascades in rice during interactions with *Xanthomonas oryzae* [103]. Expression of *WRKY3* and *WRKY4* was induced by stressful conditions generated by liquid infiltration [104]. *RPS4B* gene on MQTL2P.1 (chromosome 5) encodes disease resistance (R) protein that specifically recognizes the type III effector AvrRps4 of *P. syringae*, forming a functional complex and mediating the hypersensitive response [105]. The UDP-glycosyltransferase has been shown to have a role against *Fusarium* head blight in wheat [106]. A cluster of these genes was found on MQTL2P.1 (Table S3).

Two MQTLs were identified governing resistance against anthracnose and halo blight diseases. We detected MQTL5P.1 on chromosome 5 of common bean containing the genes *PHAVU_005G035300g* and *PHAVU_005G035500g*, which are orthologues of the *Arabidopsis* gene *BGLU42. BGLU42* is a novel component of the induced systemic resistance (ISR) signaling pathway that displays significantly enhanced resistance against gray mold disease caused by *Botrytis cinerea*. Overexpression of *BGLU42* also conferred resistance against downy mildew and bacterial speck diseases caused by *Hyaloperonospora arabidopsidis* and *P. syringae* pv. *tomato*, respectively through ISR [107]. MQTL5P.1 hosts the orthologue of *Arabidopsis ACD6* gene, which has an Ankyrin domain playing a major role in defense response against virulent bacteria through signaling of salicylic acid-dependent cell death [108, 109, 110,111]. Based on the PRGDB database, some other CGs at this MQTL were detected (Fig. 6a). Further investigations are required to validate the function of some important CGs *i.e. PHAVU_008G014700g* on MQTL8P.1 (chromosome 8) which contains nucleotide-binding site leucine-rich repeat (NB-LRR) domain implicated in plant defense interactions. As for the specific MQTLs, the MQTL10P.1 located on chromosome 10 was involved in resistance against halo blight disease. Three MQTLs *i.e.* MQTL1P.1, MQTL7P.1 and MQTL9P.1 located on chromosomes 1, 7 and 9 respectively, associated with withe mold resistance, while MQTL7P.2 located on chromosome 7 is associated with resistance to anthracnose. Hence, these MQTLs could be considered for breeding common bean towards halo blight, white mold and anthracnose resistance.

Halo blight can result in severe damages to crops when exposed to high rainfall, wind and mild temperatures [110]. In this study, MQTL10P.1 was found on chromosome 10, which contains key defensive orthologue genes like DLO family, *LOX3*, *NUP107* and *WRKY46*, which could be used to breed common bean lines to be resistant against halo blight disease. The MQTL10P.1 located on chromosome 10 contained orthologue of DLO gene family, which belongs to a group of defensive negative regulators. *DMR6* and *DLO1* mutants showed enhanced resistant to downy mildew caused by an oomycete pathogen due to their increased level of salicylic acid [112]. Lipoxygenases (LOX) family encodes lipoxygenase, which is well known for its important role in pathogen-induced defense mechanisms in different crops including potato, tomato, and common bean [113, 114]. Common bean uses the LOX to synthesize oxylipins in response to different stresses. *LOX3* is a gene on MQTL10P.1 (chromosome 10) implicated in jasmonic acid biosynthesis, which is important in inducing PR proteins that have a role in defense against plant pathogens [115]. On MQTL10P.1, orthologue of *NUP107* gene was found which is required for the plant’s response to infection by virulent pseudomonads. Further, Nup107-160 members have been predicted in the *Arabidopsis* genome to play a role in disease resistance [116]. Another key orthologue gene on MQTL10P.1 is *WRKY46*, which is associated with increased basal plant defense in *Arabidopsis* [117].

Three MQTLs *i.e.* MQTL1P.1, MQTL7P.1 and MQTL9P.1 located on chromosomes 1, 7, and 9, respectively, were specifically associated with white mold resistance. The function of orthologue of *Arabidopsis ZAR1* gene in common bean (*PHAVU_007G032100g*) on MQTL7P.1 is to mediate recognition of AvrAC, which induces effector-triggered immunity (ETI) in plant against *X. campestris* [118]. Furthermore, the gene *ZAR1* is required for recognition of HopZ1a, a *P. syringae* type III secreted effector in *Arabidopsis* [119]. The orthologues of *WRKY54*, *WRKY70* and *WRKY55* genes located on MQTL9P.1 (chromosome 9) act as positive regulators of SA-mediated defensive signaling in *Arabidopsis* [120]. The MQTL7P.2 located on chromosome 7 was predicted to be specifically involved in resistance to anthracnose. Anthracnose is a major disease of common bean causing significant economic loses to the crop in tropical regions, especially when the fruiting stage is attacked [121]. The *RIN13* gene on MQTL7P.2 is a plant defense gene that encodes RPM1-interacting protein 13, which is a nuclear-localized protein. Overexpression of *RIN13* leads to autoimmunity with high accumulation of salicylic acid, fundamental expression of pathogenesis-related genes, and enhanced resistance to plant diseases [122]. *RboHB* is also related to plant’s defense against pathogens; which is encoded by *At1g09090* and is involved in resistance against nematode. *RBOHB* gene encodes respiratory burst oxidase homolog protein B and calcium-dependent NADPH oxidase that generates superoxide [123]. *RBOHB* gene is on MQTL7P.2 (chromosome 7) and the role of this gene in defense against plant pathogens in common bean has been proven in the previous study [123]. RBOHB is a multigene family containing nine members (RBOHB A–I) in common bean. Several recent studies reported that RBOHs participate in signaling pathways of plant-pathogen interactions as well as plant-symbiont interactions [124, 125].

In conclusion, the data provided in this study revealed a number of genomic regions associated with resistance against bacterial and fungal diseases in common bean. The genomic positions of MQTLs are mainly located at sub-telomeric regions where higher gene density was also reported in common bean genome. Uneven distribution of QTLs across common bean genome was observed where the lowest number of QTLs was on chromosome 11 (*n* = 6) and the highest number of QTLs was observed on chromosome 7 (*n* = 24). Chromosome 7 had the highest number of resistance QTLs against fungal disease, while the highest number of QTLs governing resistance against bacterial agents was found on chromosome 1. Our results revealed that MQTL2P.1, MQTL7P.1, and MQTL9P.2 had the highest number of QTLs; thus considered the promising MQTLs for further investigations and breeding programs. These results on one hand provide a comprehensive insight into the genomic repertories of common bean to combat the risk of bacterial and fungal agents. On the other hand, pave the way of further researches in developing new common bean lines against a wider range of biotic constraints under the field conditions.

## Supplementary Information

**Additional file 1: Figure S1. **Venn diagram of differential expressed genes (DEGs) derived from four independent studies and the CGs located in MQTL regions (Venn diagram was drawn using a tool in this website: http://bioinformatics.psb.ugent.be/webtools/Venn/). Detailed information is presented in the supplementary Table S5.

**Additional file 2: Table S1:** The list of projected QTLs to the reference maps. QTLs related to the MQTLs were highlighted.

**Additional file 3: Table S2. **The list of CGs and all annotated genes located at each MQTL interval in this study.

**Additional file 4: Table S3.** The Arabidopsis orthologue genes in common bean located at each MQTL interval.

**Additional file 5: Table S4. **The Arabidopsis and soybean orthologue genes of common bean located at each MQTL interval.

**Additional file 6: Table S5. **List of all genes involved in the KEGG enrichment analysis.

**Additional file 7: Table S6. **List of all R-genes in evaluated MQTL regions identified in this study.

**Additional file 8: Table S7. **List of common genes between DEGs studies and current study.

## Data Availability

Not applicable. There is no data in the manuscript that need to be deposited in the public databases. There is no relevant accession number.
