# Supplementary material for: Genome-wide meta-QTL analyses provide novel insight into disease resistance repertoires in common bean
Source: BMC Genomics. 2022 Oct 3;23:680. doi: 10.1186/s12864-022-08914-w (PMC9531352; doi:10.1186/s12864-022-08914-w)
Supplement: Supplementary file 1 — Additional file 1: Figure S1. Venn diagram of differential expressed genes (DEGs) derived from four independent studies and the CGs located in MQTL regions (Venn diagram was drawn using a tool in this website: http://bioinformatics.psb.ugent.be/webtools/Venn/). Detailed information is presented in the supplementary Table S5. [file 12864_2022_8914_MOESM1_ESM.pdf]

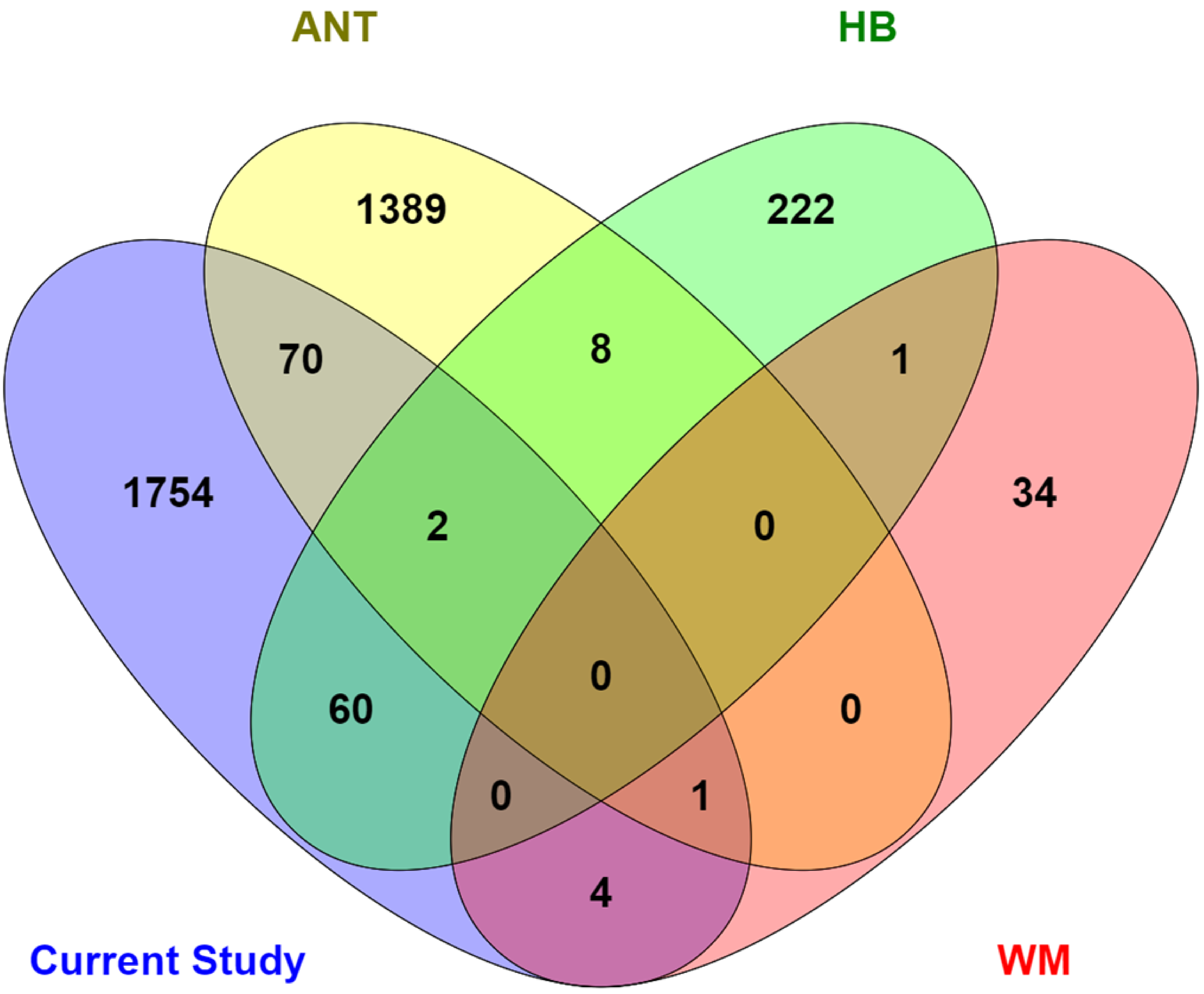

Figure S1: Venn diagram of differential expressed genes (DEGs) derived from four independent studies and the CGs located in MQTL regions (Venn diagram was drawn using a tool in this website: http://bioinformatics.psb.ugent.be/webtools/Venn/). Detailed information is presented in the supplementary Table S5.
